# A SONAR report on Nirmatrelvir/ritonavir-associated rebound COVID-19: Using new databases for evaluating new diseases

**DOI:** 10.1371/journal.pone.0308205

**Published:** 2024-09-25

**Authors:** Charles L. Bennett, Joseph Magagnoli, Krishna Gundabolu, Peter Georgantopoulos, Akida Lebby, Gretchen Watson, Kevin Knopf, Linda Martin, Kenneth R. Carson, William J. Hrushesky, Chadi Nabhan, Edward Zyszkowski, Edward B. Smith, Robert Peter Gale, Steven T. Rosen

**Affiliations:** 1 Department of Southern Network on Adverse Reactions (SONAR), University of South Carolina, Columbia, South Carolina, United States of America; 2 Clinical Pharmacy and Outcomes Sciences, College of Pharmacy, University of South Carolina, Columbia, South Carolina, United States of America; 3 Division of Hematology/Oncology, Department of Internal Medicine, Omaha School of Medicine University of Nebraska, Omaha, Nebraska, United States of America; 4 Hematology/Oncology, Highland Hospital, Alameda Health System, Oakland, California, United States of America; 5 Division of Hematology and Oncology, Department of Medicine, Feinberg School of Medicine Northwestern University, Chicago, Illinois, United States of America; 6 Centre for Haematology Research, Department of Immunology and Inflammation, Imperial College London, London, United Kingdom; 7 City of Hope Comprehensive Cancer Center and the Beckman Research Institute, Duarte, California, United States of America; Icahn School of Medicine at Mount Sinai Department of Pharmacological Sciences, UNITED STATES OF AMERICA

## Abstract

**Introduction:**

In May 2022, the Centers for Disease Control and Prevention disseminated an alert advising that “a few” persons with Nirmatrelvir/ritonavir (NM/R)-associated rebound of COVID-19 infection had been identified. Three case reports appearing as pre-print postings described the first cases. Analyses in March 2023 by NM/R’s manufacturer and the Food and Drug Administration (FDA) reported no association between NM/R and COVID-19 rebound in a large phase 3 randomized clinical trial. Our study evaluated if social media databases or electronically disseminated new articles might provide insights related to the putative new toxicity, NM/R-associated COVID-19 rebound.

**Methods:**

Information on NM/R-associated COVID-19 rebound cases was abstracted from preprint postings of non-peer-reviewed manuscripts, social media websites, electronically disseminated print and television media reports, a new FDA adverse event database for drugs that received Emergency Use Approval, and news articles in scientific journals.

**Results:**

Thirty-five persons experienced presumed or documented NM/R-associated COVID-19 rebound, based on information described in preprint services (n = 27), Twitter postings and related news articles (n = 7), and news articles without related Twitter reports (n = 1). These reports included information on dates of initial COVID-19 illness and rebound onset, COVID-19 testing, vaccine status, presentation, and outcome. A new FDA safety database identified 12,500 possible cases of this toxicity, but the quality of these data was poor. Preprint postings preceded peer-reviewed publications describing the same cases by four months. Social media websites including Instagram, Reddit, YouTube, the Center for Disease Control and Prevention’s (CDC) Health Alert Network, CDC Twitter, and Facebook did not provide clinically meaningful information on individual cases.

**Conclusion:**

Preprint services and Twitter facilitated identification of the largest case series of NM/R-associated COVID-19 rebound. The cases were reported in non-peer-reviewed media several weeks prior to the first peer-reviewed electronically disseminated publication of one person with this diagnosis.

## Introduction

In the ongoing development of COVID-19 treatments and vaccines, it is crucial to promptly recognize potential adverse effects. Our Southern Network on Adverse Reactions (SONAR) pharmacovigilance program plays a key role in this process. SONAR, funded by the state of South Carolina and the National Cancer Institute, focuses on identifying and reporting clinical information related to serious adverse drug reactions in hematology, oncology, and, since 2020, COVID-19 [1]. In a recent investigation, we utilized Twitter reports and examined the US Centers for Disease Control and Prevention’s (CDC) Vaccine Adverse Event Report System (VAERS) to identify the first fatality related to Vaccine-Induced Thrombocytopenic Thrombosis (VITT) among young women who had received the Ad26.COV2.S COVID-19 vaccine [2].

In this update, we expand the scope of SONAR to include newer data sources developed since 2000 including pre-print services, social media, a database for side effects of drugs with Emergency Use Authorization from the US Food and Drug Administration (FDA), and the CDC’s Health Alert Network which augment older databases of electronically disseminated news articles (primarily identified through Google). This expansion aims to identify potential new toxicities associated with Nirmatrelvir/Ritonavir (NM/R)-associated COVID-19 rebound [3–20].

NM/R, an oral antiviral granted Emergency Use Authorization (EUA) in December 2021, demonstrated efficacy in reducing risks of severe illness, hospitalization, and death for individuals 12 years of age or older with mild-to-moderate COVID-19 who had not been vaccinated or boosted. The CDC first reported Covid Rebound in May 2022, describing vaccinated and boosted individuals who developed symptomatic COVID-19 despite prior antigen negativity after NM/R treatment.

While CDC publications from late 2022 highlighted a "few NM/R COVID-19 rebound" cases, a combined analyses by the FDA and Pfizer of a randomized double-blind clinical trial did not establish a clear association between NM/R treatment and COVID-19 rebound. Our analysis allows for early identification of NM/R-associated COVID-19 rebound before peer-reviewed manuscripts were published. This initiative investigates the value of analyzing newer data sources in conjunction with older data sources to facilitate early detection of adverse events associated with a syndrome, NM/R-associated COVID-19 rebound, that was first identified in 2022.

## Background and methods

We analyzed datasets containing non-peer-reviewed material describing NM/R-associated presumed or documented COVID-19 rebound cases. The search period was from the time the FDA issued an EUA in December 2021 to the time of the first peer-reviewed publication of one case of NM/R-associated COVID-19 rebound on June 20, 2022 (as an e-publication). The first data set included cases described in postings on three major pre-print websites- Research Square, medRxiv, or a PMC COVID-19 pilot project that is hosted by Elsevier Incorporated (Tables 1 and 2). The second data set included social media postings on Twitter (for cases and their spouses described on social media on Twitter (now X), and electronic news sites postings (for CNN News, NBC News, New York Times, Associated Press, NPR, Washington Post, Wall Street Journal, JAMA News, San Francisco Chronicle, the Daily Mail, LA Times, CDC’s Health Alert Network, and CDC’s Health Advisory Communications) using search terms “Paxlovid,” “NM/R” and “Covid Rebound” (Tables 3 and 4). Abstracted data included information on COVID-19 vaccine, booster status, symptoms, COVID-19 antigen status, time from COVID-19 positive test to negative tests, time from negative to second positive COVID-19 test (for documented “Covid Rebound” cases only), NM/R treatment days, days from NM/R discontinuation to COVID-19 diagnosis and/or COVID-19 positive polymerase chain reaction (PCR) test, date of NM/R re-initiation (if given), and outcome. NM/R-associated rebound was either presumed (Covid-19 antigen positive test at time of rebound) or documented (COVID-19 viral identification at time of rebound). Each of these cases had a positive antigen or PCR test at the time of the initial COVID-19 infection, a negative antigen or PCR test subsequently, and then either a positive antigen or PCR test at the time of rebound (presumed case) or virally genotyped COVID-19 at time of rebound (documented case). The case report form was validated in our prior analysis of VITT [2].

**Table 1:** Nineteen NM/R-associated COVID-19 rebound cases w/ whole genome sequences (Research Square: April/May 2022 (3 cases), May 2022 (1 case)), medRxIV (May 2022 (6 cases), May 2022 (3 cases), and June 2022 (6 cases)).

Patient age range: 33–74 years median age, 59 years 7 males, 11 females.	Vaccine 100% vaccinated x2; boosted x2 in 18; boosted x3 in 1	Initial COVID-19 Dx and NM/R Rx (5 days)	Relapse COVID-19 COVID-19 PCR +, # of days past NM/R completion Range: 4–9 days post NM/R completion; median: 3 days post NM/R completion	F/up-100%: COVID at follow-up 1 patient hospitalized for 3 days	Data Source for the pre-prints- reference numbers	Data source for the peer-reviewed publication (Reference number) Dates of pre-print and peer-reviewed publication); median number of days from pre-print to peer-reviewed publication
1–71, M	Boosted X1; vaccine x2	Ag+: Day 0; NM/R day 0–4 no Sx’s day 2 to 8;	Day 9-runny nose, sore throat; asthma; OmicronBA.1.20 subvariant (d1-11) `	COVID-19 antigen test- (days 19 to 35) Omicron BA.1.20 subvariant days 1,3,5,7,9,11	49.50	8 (April 26, 2022- Sept 7, 2022 (105 days))
2–69, M	Boosted X2; vaccine x2	Ag+ days 0 to 3; Sx’s days 0 to 3; NM/R day 1–5; Ag-: days 4 to 9; 2 neg PCR tests	Day 10- mild Sx’s x 3 days;	Sx’ day 0 to 4; returned on day 10; COVID 19 Ag neg on days 4–8; 12–18; Omicron BA.2.9 subvariant days 2, 10	50	8 May 13, 2022- Sept 7, 2022 (85 days)
3–50, F housemate of case 2	Boosted X2; vaccine x2	Day 0–3 Sx’s; COVID-19 Ag + on days 2 to 5; NM/R days 3–7	Day 10 COVID-19 AG-positive days 6–8; Antigen+: days 9 to 11	Omicron BA.2.9 subvariant days 2,4, and 9; Ag negative- days 12–18	49	8 (April 26, 2022- Sept 7, 2022 (105 days))
4-Not stated	Boosted x 2; vaccine x 1 12/20/2021	Day 0–4 NM/R; Sx’s resolved	Day 9: Sx’s: fatigue, sore throat. SARS-CoV2 isolated w/ Sx’s	Not stated.	50	8 (May 13, 2022- June 20, 2022 (38 days))
5-,M, age between 33 and 74 yrs	Boosted x 1; vaccinated x 2	NM/R Rx 5 days, started between days 1–4	Between 3–9 days after completing NM/R Rx; Viral sequences excluded reinfection or mutation	Not stated	51	20 (June 17, 2022- Oct 6, 2022 (80 days))
6-M, age between 33 and 74 yrs	Boosted x 1; vaccinated x 2; + comorbidity	NM/R Rx 5 days, started between days 1–4	Between 3–9 days after completing NM/R Rx; Viral sequences excluded reinfection or mutation	Not stated	51	20 (June 17, 2022- Oct 6, 2022 (80 days))
7-M, age between 33 and 74 yrs	Boosted x 1; vaccinated x 2; + comorbidity	NM/R Rx 5 days, started between days 1–4	Between 3–9 days after completing NM/R Rx; Viral sequences excluded reinfection or mutation	Not stated	51	20 (June 17, 2022- Oct 6, 2022 (80 days))
8-F, age between 33 and 74 yrs	Boosted x 1; vaccinated x 2; + comorbidity	NM/R Rx 5 days, started between days 1–4	Between 3–9 days after completing NM/R Rx; Viral sequences excluded reinfection or mutation	Not stated	51	20 (June 17, 2022- Oct 6, 2022 (80 days))
9-F, age between 33 and 74 yrs	Boosted x 1; vaccinated x 2; + comorbidity	NM/R Rx 5 days, started between days 1–4	Between 3–9 days after completing NM/R Rx; Viral sequences excluded reinfection or mutation	Not stated	51	20 (June 17, 2022- Oct 6, 2022 (80 days))
10-F, age between 33 and 74 yrs	Boosted x 1; vaccinated x 2; + comorbidity	NM/R Rx 5 days, started between days 1–4	Between 3–9 days after completing NM/R Rx; Viral sequences excluded reinfection or mutation	Not stated	51	20 (June 17, 2022- Oct 6, 2022 (80 days))
11–31, F	Vaccine x 2, boosted x 2	NM/r- days 0–5; Rapid ag:—on day 6; Sxs: day 0–4	Sx’s: days 8–24 Rapid Ag+: day 10	Day 20 PCR test: -	52	15 (50 days) May 26, 2022- e-print on July 15,2022
12–55, M	Vaccine x 2, Boosted x1	Sx: days 0–1 Rapid Rapid Antigen test: neg day 6	Sx’s: days 10–24 Viral load + (day 10–18) Rapid ag test: + day 10	PCR: neg on day 23	52	15 (50 days) May 26, 2022- e-print on July 15,2022
13–47, F	Vaccine x 2, boosted x 1	Sxs: days 2–4 NM/r: days 1–5 Radid test:—on day 6	Rapid Ag: + day 9; Sx’s: Days 12–20; Viral load: +: days 16–22	PCR:—on day 23	52	15 (50 days) May 26, 2022- e-print on July 15,2022
14–52, F	Vaccine 2, boosted x 3 Immuo-suppressing condition	NM/r: day 0–4; Sx’s: day 0; Rapid Ag test–on day 6	Rapid Ag test: + on day 9; Sx’s: day 8- day 12; Viral load: + on days 10–16	PCR:—on day 18	52	15 (50 days) May 26, 2022- e-print on July 15,2022
15–48, F	Vaccine x 2, Boosted x 2	NM/r: days 0–4; Sx: days 0–4; Rapid Ag test–(not reported):	Sx’s: days 8–20; Rapid Ag test: + on day 10; culture + on days 10 and 11	PCR:—on day 16	52	15 (50 days) May 26, 2022- e-print on July 15,2022
16–64, F	Vaccine x 2, Boosted x 1	NM/r: days 0–5; Sx’ days0-5; Rapid Ag test:—on day 6	Rapid Ag test: + on day 11; Sx’s: days 12–24; Viral load: + days 12–17	PCR:—on day 19	52	15 (50 days) May 26, 2022- e-print on July 15,2022
17–55, M (with RA, vasculitis)	Vaccinated x 2; boosted x 2	Sx’s began: day 1; Sx’s improved: days 5 to 7 NM/R: days 2 to 6; Lateral flow test (LFT): + day 1	Sx’s: Day 8 Lateral flow test+: day 6	NA	53	19(26 days) May 22, 2022- June 17, 2022
18–44, F (with Crohn’s disease)	Vaccinated x 2; boosted x 2	Sx’s began: day 1; Sx’s improved: days 3 to 5 NM/R: days 4 to 9; Lateral flow test: + days 1–3	Sx’s: day 14 Lateral Flow Test+: days 5 to 11	NA	53	19(26 days) May 22, 2022- June 17, 2022
19–59, F (with vasculitis)	Vaccinated x 2; boosted x 2	Sx’s began: day 1; NM/R: days 2 to 7; Lateral flow test: + day 1	Sx’s: 19 Lateral Flow Test+: day 6	NA	53	19(26 days) May 22, 2022- June 17, 2022

**Table 2:** Eight anonymous persons with presumed NM/r-associated COVID-19 rebound published by pre-print services -: (medRxiv (1 case) and Research Square (7 cases).

Case #	Patient Median age: 64.5 (range 53–81 yrs); 50% F	Vaccine	Initial COVID-19 Dx and NM/R Rx	Relapse COV ID-19 PCR # of days s/p NM/R Rx	F/up-COVID	Data Source (pre-print)	Data Source Reference Number (date from pre-print to peer-review publication) number of days to publication
1-MGH-7	Not stated	Vaccine X2; boost X1	NM/r: Days 0–4; Sx’s: days 0–2 Rapid Ag:—on day 4	Sx: days 10–20 Rapid Ag: + on day 12; Culture + days 14–16	Culture neg: day 19 Sx’S neg day 20 on	52	15 May 26, 2002- June 23, 2022 (28 days) (e-print)
2-R-square-patient 4	63 years, male;	Vaccine x 2; 1 boost (6 months prior)	Sx’s: days 0–6: NM/r: days 1–5: Ag + day 2; PCR+: day 3	Sx’s: day 11–13; Covid-19 antigen+: day 13; 1 transmission event–day 10	No Sx’s: Days 14–22:	50	8(May 13, 2022- Sept 7, 2022) (75 days)
3-R-square-patient 5	31 years, female	Vaccine x 2; boost x1 (6 months prior)	NM/r: days 1–4; Sx’s: days 0–6; Ag +: days 0 and 4; PCR +: day 2	Sx’s: Day 9–14; Antigen test: + on days 8, 12, and 14	Mild Sx’s: days 15–22; PCR- day 16	50	8 (May 13, 2022- Sept 7, 2022 (75 days)
4-R-square-patient 6	34 years, male	Vaccine x 2; boost x 1 (6 mos prior)	NM/r: days 1–5; Sx’s: days1-4; Ag +: day 0: PCR +: day 1	Mild Sx’s: days 13–16; Antigen +: days 15 and 16	No Sx’s- days17- 22; Antigen -: day 18	50	8 (May 13, 2022- Sept 7, 2022 (75 days)
5-R-square-patient 7	47 years, female	Vaccine x 2; boost x 1 (4 mos prior)	NM/r: days 1–5: Sx’s: days 0–3; Antigen +: day 0	Sx’s: days 11–17; Antigen +: Days 13 and 16:	Mild Sx’s: days 17–21; Antigen +: day 16; Antigen -: day 18	50	8 (May 13, 2022- Sept 7, 2022 (75 days)
6-R-square-patient 8	55 years, male	Vaccine x 2; boost x 1 (4 mos prior)	NM/r: days 0–4; Sx’sdays 0–1; mild sx’s: days 2–6;	Sx’s: days 10–13; mild Sx’s: days14- 16;; PCR +: days 11, 12, 14, and 16	No Sx’s: days 17–22; PCR-: days 18 and 20	50	8 (May 13, 2022- Sept 7, 2022 (75 days)
7-R-square-patient 9	67 years, male	Vaccine x 2; boost x 2 (5 mos prior)	NM/r: days 2–6: Sx’s days 0–1; mild Sx’s” 2 and 3; Antigen+: day 2	Sx’s: days 12–15; Antigen +: days 13 and 16; transmission- day 12	No Sx’s: days 15–22; Antigen-: day 22	50	8 (May123, 2022- Sept 7, 2022 (75 days)
8-R-square-patient 10	68 years, female	Vaccine x 2; boost x 2 (1 mo prior)	NM/r: days 2–6: Sx’s- mild–days 0–3;Antogen+: day 2	Sx’s: days 11–16; Antigen +: days 10–15	No Sx’s: days 14–17; antigen-: day 18	50	8 (May 13, 2022- Sept 7, 2022 (75 days)

**Table 3:** Eight persons with presumed NM/R-associated COVID-19 rebound described in public non-medical sources.

Case #- date PCR+ Median	Patient Age range, 50 to 81 years (median age, 64 years); 50% female	Vaccine 100% vaccinated x 2/ boosted x 2	Initial COVID-19 Dx and NM/R Rx 100% received 5 days of NM/R Rx	Relapse COV ID-19 PCR +, # of days past NM/R Rx Median onset day 4 after NM/R completed (days 3–19)	F/up-COVID 0% hospitalized; 1/8 developed possible “long COVID”	Date of Twitter–Date of Public News Notification (median of 0.5 days, range 0 to 8 days)
1; 5/8	65, F, wife of the Chairman of Medicine’ of a medical school,	Full-2/boosted x 2	5/8- Sx 5/9- NM/R 5/9- NM/R	Date not stated-Rebound	Long-COVID	(May 16, 2022- May 24, 2022) (8 days)
2; 5/2; PCR-pos; PCR neg: 5/8 and 5/9	50, F, Associate Professor of Oncology, Medical School on the East Coast of the US	Full + boosted x 2	5/2-Sx 5/2 PCR+ 5/2–5/6: 5 day NM/R	5/16 (day 10)	5/ 27 (Day 28): neg COVID-19 Rapid Antigen Test	(May 17, 2022- May 24, 2022) (7 days)
3; 5/12-PCR+	64 M, Dean of a School of Tropical Medicine	Full + boosted x 2	May 12–5 day NM/TR started	May 17 (day 5) with Sx’s- just after completing NM/R (day 5)	May 22- (day 5)- PCR-neg	(May 17, 2022- May 22, 2022) (5 days)
4; 7/22 Sx and PCR+	79, M, Senior executive in the Biden Administration	Full + boosted x 2	7/22 Sx and 7/22 PCR+; NMR 7/22-7/27	7/30 -PCR+’; 7/30- no Sx’s; 7/30 no NM/R Rx (day 3)	8/ 8-8/9- PCR- day 16 and day 17	(July 30, 2022- July 30, 2022) (0 days)
5; 8/16 Sx’x and PCR+	71, F Wife of senior executive in the Biden Administration	Full + boosted x 2	8/16 PCR+; NM/R x 5 days	8/20 + 8/21- PCR- 8/24 (day 3)-aSx rebound;	8/29- PCR-	(August 25, 2022- August 25, 2022) (0 days)
6: 6/15- Sx’s; PCR+;NM/R	81,M, Former Director of a NIH Institute	Full + boosted x 2	6/15- mild Sx’s; PCR+’ NM/R x 5	6/19- PCR+ (day 4); Sx’s; NM/r Rx x 5 days (2^nd^ course)	6/20 PCR+ 6/21- PCR-	(June 29, 2022- June 30, 2022) (1 day)
7: 6/22- PCR+; NM/R started; mild Sx’s	53,F, Executive at the Centers for Disease Control and Prevention (CDC)	Full + boosted x 2	10/22 PCR+ NM/R Rx x 5	10/30- PCR+; (day 8)		(no Twitter release- CDC News Release on Oct 31, 2022- NBC News on Oct 31, 2022) (0 days)
8–4/22; Sx’s; NMR 5-day started	57, M, television talk show host	Full + boosted x 2	4.22- Sx’s PCR+; NM/R x 5 days	Relapse COVID-19 on 5/9/; Sx’s; PCR + (day 19)	COVID- on 5/15	(May 9 (Twitter)- announces rebound COIVID-19- May 9,2022 (0 days)

**Table 4:** 21^st^ Century datasets utilized in evaluating NM/R-associated COVID-19 rebound [44–48].

Database- start date of the database	# of users	# of cases reported on the specific web site	Completeness of individual case reports	Information on Omicron versus Delta	Case information subsequently reported in the peer-reviewed literature	Descriptions of specific patients	Updated
Google -1998 [44]	246 million	4	Intermediate	None	NEJM-1 pt (the former director of NIAID)	Virologist, Executive in the Biden Administration, wife of the executive in the Biden Administration, CDC Executive, Host of a Television Talk Show	Every second
Facebook-2004 [45]	190 milion	0	Low	…	…	…	Every second
REDDIT-2005 [48]	52 million	0	Low	…	…	…	Every second
Twitter-2006 [46]	63 million	3	Moderately High	None	No	Wife of Chairman of Medicine at a medical school, Dean at a graduate school of tropic medicine, Oncology Associate Professor at a medical school	Every second
CDC-Twitter- 2008	5.3 million	0	….	…	…	…	Daily
Instagram-2010 [47]	143 million	0	Low	…	…	…	Every second
FAERS Dashboard-2021	Not known	12,688	Very low	Very little	Not known	…	Updated weekly;
Pre-print services	# of pre-prints		Quality of case information	Demographics	# of COVID-19 rebound patients	Information on individual patients w/ confirmed or suspected NM/r rebound	Where from
medRxiv- 2018			High	No	7 pts	7 pts (6 confirmed and 1 presumed COVID-19 NM/r rebound	7 pts from Mass General Hospital study
Research Square-2018	150,000	3	High	Yes	10 pts	10 pts (3 confirmed and 7 presumed NM/r patients)	2 from Columbia; 1 from New England VA; 7 from Colombia U study
NIH pre-print on PMC site– 2020		0	NA	NA	Nas	NA	NA

We reviewed adverse event report information contained in FDA Adverse Event Reporting System’s (FAERS) Dashboard Database for Drugs that received EUA. We also reviewed case information on adverse reaction(s), seriousness, outcomes, gender, adverse event date, age, and medication manufacturer.

We used the key words “COVID-19,” “Paxlovid,” and “rebound” to identify and then analyze reports of NM/R-associated COVID-19 rebound which had been described on Google. Searches identified information in electronically posted news media reports. Abstracted information included media name, name of the person with COVID-19 and NM/R rebound, and the same elements evaluated in earlier disseminations on Twitter.

Two research assistants independently abstracted relevant data. Concordance was > 95%. Discrepancies were adjudicated by the Principal Investigator. Because the manuscript included no identifiable human information, it was designated as Expedited Human Subjects review (University of South Carolina IRB ID: Pro00132017).

## Results

Overall, 35 persons with presumed or documented NM/R-associated COVID-19 rebound illnesses were identified. Their median age was 53 years (range, 31 to 81 years), 45.5% were male with and in two instances gender was not provided. All 35 individuals had received at least two COVID-19 vaccinations and almost all had received an additional COVID-19 booster. NM/R treatment was for 5 days, beginning generally with the onset of symptoms and a positive PCR COVID-19 antigen test on either day 0 or 1. Rebound symptoms began a median of day 9 days after COVID-19 outset (range, 4 to 19 days) and resolved at a median of day 17 (range 6 to 28 days).

### Pre-print cases (n = 27)

Among 27 NM/R-associated presumed or documented NM/R-associated COVID-19 rebound cases identified in preprint postings, investigators confirmed with whole gene sequencing techniques 19 cases of Covid-19 rebound. Among these cases the median age was 47.5 years (range, 31 to 71 years), and 38.9% were male (Table 1). Pre-print sources for these 19 cases included Research Square (4 cases published in April and May 2022) and MedRxIV (15 cases published in May and June 2022). Detailed case information on all of these 19 cases was subsequently published as an e-Correspondence in the New England Journal of Medicine in September 2022 (for three cases reported initially in Research Square), two electronic publications in Clinical Infectious Diseases (in June 2022); for 14 cases reported initially in MedRxIV), and Journal of Infection (in October 2022 for three cases reported initially in MedRxIV). Three pre-print publications were posted between April 1 and June 7, 2022 [3–5]. The related three peer-reviewed publications appeared about 50 days later as two electronically disseminated peer-reviewed pre-prints in the journal Clinical Infectious Diseases or as one peer-reviewed e-correspondence in the New England Journal of Medicine [6–10]. Of note, publication of the New England Journal of Medicine (NEJM) e-Correspondence on September 7, 2022 occurred after the authors responded to two rounds of comments from peer-reviewers and the editor [8]. The e-Correspondence was initially submitted on May 12, 2022- one day before version 2 of the pre-print was posted and 11 days before the version 3 pre-print was posted [8]. Initial revisions were requested on June 2 and submitted on June 6. The second round of revisions was requested on June 20 and submitted on June 23. The final version of the e-Correspondence was accepted on July 13, 2022 and appeared in the print journal on September 7, 2022 [8]. The three pre-print versions of data from Charness, Carlin, and others that preceded the NEJM e-Correspondence were viewed 21,817 times and were downloaded 1400 times.

For these 19 individuals with confirmed NM/R- associated COVID-19 rebound, the rebound occurred about 10 days after the initial COVID-19 infection and 5 days after each individual had completed five days of NM/R treatment, involved persons of all ages, few persons had pre-existing comorbid illnesses or immunodeficiencies, and all 19 cases of confirmed NM/R-associated COVID-19 rebound survived and were not hospitalized. All 19 cases had been vaccinated twice and boosted twice against COVID-19. Most patients with documented NMN/R-associated COVID-19 rebound had no symptoms at the time of relapse. Longer- term information on the 19 cases was not reported.

An additional eight cases of presumed NM/R-associated COVID-19 rebound with viral PCR testing or rapid antigen testing were published in pre-print publications. The median age of these individuals was 55 years (range, 31 to 68 years), and 57.1% were male (Table 2). Specific pre-print websites were medRxIV (1 case) and Research Square (7 cases). Three cases included in the version 1 Research Square pre-print publication NM/R-associated COVID-19 cases were also referred to in a May 2002 CDC Health Alert about NM/R-associated COVID-19 rebound. All 8 cases of presumed NM/R-associated COVID-19 rebound described in Table 2 had presented with mild symptoms, usually an upper respiratory infection, at around day 10 after the initial infection and 5 days after completing a 5-day NM/R regimen. All 8 cases survived and were not hospitalized. Longer-term information on the cases was not reported.

### Social media and electronic media (n = 8 cases)

Review of posts on Twitter identified seven individuals with presumed NM/R-associated COVID-19 rebound and electronically disseminated news articles described these same persons as well as one additional person with presumed NM/R-associated COVID-19 rebound [21–36]. The median age of the eight individuals was 64.5 years (range 50 to 81 years), and 50% were female (Table 3). In each instance individuals took NM/R for the prescribed time of five days. In two instances reports included information on the number of days before presumed “Rebound COVID-19” was diagnosed.

The first description in the United States of persons with possible COVID-19 NM/R -rebound was disseminated in social media in February 2022. Clinical information was not included in this report and therefore a diagnosis of presumed or documented NM/R-associated COVID-19 rebound could not be made. Overall, social media COVID-19 NM/R rebound case information differed amongst the four major social media sites. Many individuals reported to Facebook that NM/R provided relief from COVID-19 symptoms, that they would take NM/R again, and sought to inform others about potential NM/R downsides. YouTube and Instagram social media provided very little information about clinical symptoms among persons with COVID-19 rebound. Reddit had pretty complete information on patient symptoms. Overall, data completeness was poor for all cases reported in social media, except for Twitter reports from three physicians (Robert Wachter MD (describing his wife’s case), Peter Hotez MD PhD (describing himself), and Tatiana Powell MD (describing herself) and additional Twitter (now known as X) reports describing clinical conditions of famous individuals including President Joseph Biden, First Lady Jill Biden PhD, former Centers for Disease Control and Prevention Director Rochelle Walensky MD MPH, former Chief Medical Advisor to the President of the United States Anthony Fauci MD, and late-night television talk show host Stephen Colbert.

Detailed case information on Twitter for famous persons as well as in related electronic news releases was reviewed. Clinical information for three persons whose case information was initially disseminated on Twitter (now known as X) by three physicians who frequently report on COVID-19 issues was also reviewed. Two of these three physicians (Peter Hotez MD PhD, dean of the National School of Tropical Medicine at Baylor College of Medicine and Tatiana Powell MD, an associate professor of oncology at the Johns Hopkins University School of Medicine) described their own clinical and laboratory findings when each of these physicians developed of presumed NM/R-associated COVID-19 rebound and the third case, Katie Wachter, was the wife of Robert Wachter MD, chair of the University of California, San Francisco’s Department of Medicine (Table 3). Each of the cases had initially recovered from COVID-19 and developed presumed COVID-19 NM/R rebound. Information for each of the famous persons identified above was disseminated both on Twitter (now known as X) and on electronic news postings.

### Details of the eight cases are described below

News article cases involving famous individuals (5 individuals). Four of these cases are also described in Twitter reports.

The then 79-year-old President of the United States, was vaccinated twice and boosted twice with the BNT1682 vaccine and developed a positive COVID-19 test and cold symptoms. He completed a 5-day course of NM/R, had negative COVID-19 tests, but experienced COVID-19 antigen-test positive NM/R rebound event three days later. He tested COVID-19 positive for 7 days with COVID-19 NM/R rebound before testing negative on day 12.

The then 71-year-old First Lady of the United States was vaccinated twice and boosted twice with the BNT1682 vaccine and subsequently developed COVID-19. She was treated with 5 days of NM/R, rendering her COVID-19 negative. She experienced symptomless COVID-19 NM/R rebound diagnosed by a COVID-19 positive antigen test and subsequently, after testing COVID-19 antigen negative for two consecutive days, she quarantined for five additional days and was not retreated with NM/R throughout the rebound episode.

The then 81-year-old former Head of the National Institutes of Allergy Immunology and Infectious Diseases, became COVID-19 positive despite two doses of COVID-19 vaccine and two booster doses of COVID-19 vaccine. He received 5-days of NM/R. He tested COVID-19 antigen negative for three days. On day four, he was COVID-19 antigen positive, COVID-19 NM/R rebound was diagnosed, and his symptoms were more intense than those that he experienced with initial infection. His physician prescribed a second five-day NM/R course. On day four of this course, he was still COVID-19 antigen positive. A few days later, he tested COVID-19 antigen negative.

The then 53-year-old former Director of the CDC tested COVID-19 antigen positive after previously receiving two doses of a COVID-19 vaccine and two COVID-19 vaccine boosters. The leader received a 5-day course of NM/R and tested COVID-19 antigen negative afterwards, but subsequently developed PCR-detected COVID-19 antigen positive rebound.

The then 57-year-old late night television talk show host, reported by Twitter that he had COVID-19 illness despite two vaccine doses and one booster. He was treated with 5-days of NM/R but developed a PCR-positive COVID-19 rebound.

Reports in news media on these five cases included complete information on vaccination status, COVID-19 testing information, and dates of starting and stopping NM/R. The information disseminated on Twitter was also disseminated on the same day in electronic news communications for four of the five famous individuals Rocehelle Walensky MD being the lone exception with her information not being disseminated in the print news media on the same day that it appeared on Twitter.

### Twitter and news media information of persons who were not nationally famous (n = 3 cases)

Twitter described case-specific information on three persons with presumed cases of NM/R-associated COVID-19 rebound. Related clinical information for each case was reported on electronic news communications disseminated by the Journal of the American Medical Association or the San Francisco Chronicle. This follow-up information appeared between five and eight days after the initial tweeted medical information.

Robert Wachter MD, a Chairman of Medicine at the University of California at San Francisco School of Medicine reported on Twitter that his wife, Katie, who had been fully COVID-19 vaccinated and boosted, had experienced COVID-19 NM/R rebound. She tested COVID-19 antigen positive on day 0. NM/R began the following day. On day 2 of NM/R, she felt better. On day 5, she completed NM/R. On day 8, she tested COVID-19 antigen negative. A few days later presumed NM/R rebound was diagnosed. At 5-weeks follow-up, her husband reported that she had lingering “brain fog” and that she was experiencing severe fatigue.

A 45-year-old oncologist at the Johns Hopkins University School of Oncology reported on Twitter that after she had been fully vaccinated and boosted, she developed symptomatic Rapid Antigen test-positive COVID-19 infection. Eight days after completing 5 days of NM/R, she was COVID-19 positive. She tweeted that she was taking a second 5-day course of NM/R. On Day 27, negative COVID-19 Rapid Antigen Test was negative.

A 60-year old Dean of a graduate school as part of medical school, described on Twitter his own case. He received five-days of NM/R for antigen-positive COVID-19 infection. Five days later, he reported developing rhinorrhea and sore throat and a had a positive COVID-19 antigen test. He took a second 5-day NM/R treatment, and his symptoms improved. His COVID-19 antigen test returned to negative.

### FAERS dashboard analysis

As of July 29, 2022, there were 4,850 de-identified FAERS reports about NM/R and disease recurrence in the new FAERS Dashboard [9, 11, 37]. In one instance COVID-19 recurrence was fatal, in four instances it was described as life-threatening, and in nine instances recurrence contributed to disability. In 45 instances individuals were hospitalized, in 88 cases outcomes of CIVID-19 recurrence were described as having outcomes other than death, life-threatening, disabled, hospitalized, or non-serious events, and 4703 instances of COVID-19 recurrence events were described as “non-serious” [37]. Information on number of days until NM/R-associated COVID-19 rebound symptoms occurred was generally missing. Disproportionality analysis showed that NM/R use was significantly associated with disease occurrence (relative odds risk (ROR): 212.01; 95% confidence interval (CI): 162.85–276.01). When restricting the FAERS analysis to 3129 reports from healthcare professionals, NM/R use was associated with even higher correlation with disease recurrence (ROR: 421.38; 95% CI: 273.60–648.99). Overall, NM/R-associated COVID-19 recurrence (“rebound” was not recorded in the FAERS reports) accounted for 40.4% of all NM/R adverse events reported to FAERS. Disproportionality analysis found that NM/R was significantly associated with disease recurrence, while no signal was detected for any of the other COVID-19 drugs including casirivimab/imdevimab, remdesivir, bamlanivimab, bamlanivimab/etesvimab, sotrovimab, baricitnib, bebltelovimab, cilgavimab/tixagevimab, and tocilizumab. Moreover, the FAERS analysis indicated that for most patients with NM/R-associated “disease recurrence” was “non-serious”. The authors concluded that the association between NM/R and COVID-19 disease recurrence should not be overlooked.

### Summary of findings for 35 well-described presumed or documented NM/R-associated COVID-19 rebound patients

A synthesis of the findings from this case series is that NM/R-associated COVID-19 rebound is likely to be more common that initially reported by the CDC, the FDA, and Pfizer; affects males and females of all ages (most of whom do not have comorbid or immunologic illnesses), presents about five days after completing a 5-day course of NM/R; and occurs among persons who had been fully vaccinated and boosted at the time of onset. Presentation was either asymptomatic among persons who were being serially evaluated with COVID-19 viral testing or included mild or moderate symptoms, usually upper respiratory infection-like symptoms. Only three individuals were retreated with NM/R. In each instance symptoms resolved within days and in no instance were individuals hospitalized. One of these three individuals with COVID-19 rebound has developed a documented long COVID syndrome. Viral genotyping analyses for 17 persons indicated that cases of rebound NMR-associated COVID-19 did not represent mutated virus, but rather they were infected with a COVID-19 virus with the same genotype as that which was identified at time of initial infection.

## Discussion

Preprint services and Twitter facilitated identification of 35 cases of NM/R-associated COVID-19 rebound which were reported prior to the first peer-reviewed publication of one case of a person with this diagnosis (S1 Table). In these 35 cases people experienced mild upper respiratory infection like symptoms, or were asymptomatic at the time of NM/R-associated COVID-19 rebound.

Twenty seven of the 35 cases were comprehensively described in pre-prints about two months before the related information was published in peer-reviewed medical journals. Of 125,000 articles published on the pandemic within 10 months of the first confirmed case, more than 30,000 were on pre-print servers [38]. COVID-19 preprints were accessed more, cited more, and shared more on on-line platforms than non-COVID-19 preprints. COVID-19 preprints have fewer words per manuscript and reviewed faster than non-COVID-19 preprints. Most pre-prints for NM/R-associated COVID-19 rebound appeared at least five months prior to peer-reviewed publications describing similar findings.

Social media, specifically Twitter (now known as X), provided information on eight cases of NM/R-associated COVID-19 rebound. Twitter cases for four famous individuals appeared in social media on the same day that electronic news media described these cases. For three additional Twitter disseminated cases describing a Chairman of Medicine’s wife, a Dean of Tropical Medicine, and an academic oncologist, about one week elapsed before electronic print media described the cases initially described on Twitter. The three authors of tweets describing these three cases did not publish case histories in peer-reviewed medical journals. Rather, each physician frequently tweeted information on COVID-19 under their name, indicating that they felt that the best way to communicate accurate health information that they had read, analyzed, or personally collected was via tweeting this information.

One recent study found that over 2,000 physicians use Twitter, with all of these individuals “tweeting” more than once per day and having more than 300 followers each [39]. Among health care providers, Twitter has increased in popularity by allowing health care professionals to reach a brand audience that includes other physicians, medical trainees, other health care professionals and patients and is an open access source of information [40–42]. Twitter allows researchers and clinicians to connect and share information about interesting case studies, such as those included in this case series. Exchange of case information on Twitter can facilitate research, such as follow-on peer-reviewed case series of persons with NM/R-associated COVID-19 rebound that were electronically published in June 2022 [43].

We acknowledge that a major concern relates to the accuracy of information disseminated on Twitter. There is no systematic way to evaluate Twitter misinformation. However, independent corroborative information on six tweeted cases involving an associate professor of oncology a large medical school, a dean of a school of tropical medicine, the wife of a chairman of medicine at another large medical school, a senior person in the Biden administration and his wife, a former director an institute of the National Institute of Health, a former Director of the CDC, and a national late night talk show host. Their circumstances were also widely reported in news articles that appeared in the Washington Post, the New York Times, and in JAMA news reports.

This pharmacovigilance initiative performed well with respect to synthesizing information on 35 persons with NM/R-associated COVID-19 rebound about reporting this information about two to three months before peer-reviewed publications describing many of these same caseswere electronically or hard-copy published in peer-reviewed medical journals.

Before the first report on NM/R-associated COVID-19 rebound appeared as an e-publication in Clinical Infectious Diseases in June 2022, news media and a preprint posting from researchers at Scripps Research Institute reported that NM/R-associated COVID-19 rebound was likely to be common. A medRXIV pre-print reporting on viral kinetics of SARS-CoV-@ Omicron infection in 36 mRNA-vaccinated individuals (11 of whom were treated with NM/R). The researchers showed that NM/R treatment was associated with greater incidence of COVID-19 viral rebound compared to no treatment.

In our study, COVID-19 rebound onset occurred on aggregate within days of completing a 5-day regimen of NM/R and rarely resulted in hospitalization or long-term sequelae. Similarly, the CDC reported that review of patient information from two randomized double-blind phase II and phase III clinical trials of adult outpatients with mild to moderate COVID-19, only 1 of 77 patients treated with NM/R experienced viral rebound [10]. This person did not require in-hospital medical attention. Only three individuals reported herein with NM/R-associated COVID-19 rebound described worse clinical symptoms with the onset of COVID-19 rebound. These three individuals received second NM/R courses. All three rapidly recovered. It was reassuring that cases identified that a single omicron COVID-19 strain was present with initial infection and with subsequent rebound which is consistent with incompletely treated rather than NM/R-resistant COVID-19 infection.

Experiences described in the case series raise concern that five days may have been too short for treating COVID-19. Pfizer has initiated a trial comparing 5-days versus 10-days of NM/R among immunocompromised persons. Also, our findings do not provide insight about the duration of time for isolation that is required after a case of NM/R-associated COVID-19 rebound occurs. The authors of one large case series reported that increases in antibody and cellular immune responses occurred during rebound compared with the acute COVID-19 presentation [20]. Also, they report that there is no evidence to support the hypotheses that humoral or cellular immune responses led to symptomatic rebounds or that resistant mutations had occurred during COVID-19 rebound [20].

Our novel approach for evaluating toxicities from COVID-19 treatments or vaccines using newer data sources augmented by older data sources facilitated early identification of NM/R-associated COVID-19 rebound in this study as well as vaccine-induced thrombocytopenic purpura and other hematologic toxicities caused by novel COVID-19 vaccine associated hematologic toxicity [2, 43]. Case information reported on Research Square preprints server were publicly disseminated two to three months earlier than the first peer-reviewed report describing these three whole genome sequenced cases of NM/R-associated COVID-19 rebound and 10 cases of assumed NM/R associated COVID-19 rebound [30]. COVID- 19 has created an increased demand for early adverse event identification that has been aided by pre-prints and social media, many of which focus on COVID-19 rebound [44–53]. SONAR is helping to meet that demand [31].

Since the beginning of the COVID-19 epidemic, daily social media usage per individual in the United States has averaged 65 minutes.

1998. **Facebook** has 190 million users in the US annually [45]. It allows its users to form groups. Once members receive access, they can interact through discussion with other members. When searching for COVID-19 adverse event discussion groups, a group entitled “Covid Vaccine–Long Haul Autoimmune Support” populated a private group with 3,600 members. The group description includes: “If you have taken the vaccine and one or more days later experienced dizziness, mental fog, heart palpitations, panic attacks, headaches, chills, weakness, tingling of skull and/or spinal cord, ER visits with perfect vitals this is the place for you”. A mean of 985 Facebook posts have occurred monthly.

2005. **Reddit** website has 53 million users annually in the US [48]. Reddit users submit content to the site that is voted up or down by other members. Posts are organized into “communities” or “subreddits”. Submissions with larger number of upvotes appear at the top of the posts.

2006. **Twitter** has 63 million users annually [46]. It allows 200 characters per “tweet.” This information can be “re-tweeted” but not modified.

2008. The **CDC Twitter** account has 4.7 million followers (versus 4.2 million Facebook followers and 2.6 million Instagram followers). The CDC broadcast on Twitter includes messages that are viewed as intentional, engaging, and informative about COVID-19. A unique aspect to this platform is that Twitter posts can appear at the top of a page. CDC has currently one post pinned with a tool that allows one to search the COVID-19 community level in their geographic area. There are guidelines detailing action that community members should take at each level including vaccination timing and mask wearing.

2010. **Instagram** has 143 million users in the US annually [47]. Its platform differs has pages but no groups. Content creators have the option to post on their feed or to add to their initial story. Feed posts remain on the profile whereas story posts disappear after 24 hours. Multiple photos can be posted in a thread, along with a caption. Discussion occurs as comments and members of the Instagram community can respond to comments from one another.

2015. **CDC’s Health Alert Network (HAN)** is CDC’s primary method of sharing cleared information about urgent public health incidents with public information officers; federal, state, territorial, tribal, and local public health practitioners; clinicians; and public health laboratories. CDC’s HAN collaborates with federal, state, territorial, tribal, and city/county partners to develop protocols and stakeholder relationships that will ensure a robust interoperable platform for the rapid distribution of public health information. The HAN messaging system directly and indirectly transmits Health Alerts, Advisories, Updates, and Info Services to more than one million recipients.

Pre-print of non-peer-reviewed manuscripts

2018. **Research Square** allows authors to share work early, gain feedback from the community, and make manuscript changes on subsequent versions of the submission prior to peer review acceptance in a journal. Over 150,000 pre-prints have been submitted to Research Square. In 2021, Research Square was purchased by Elsevier.

2018. **medRxiv** allows researchers to submit pre-prints to this site. This has been the primary site for pre-prints for COVID-19, peaking at 40% of all COVID-19 manuscripts in 2020 and now decreasing to 28% of all COVID-19 manuscripts in 2022.

**2020. In a Pilot Project by PubMedCentral (PMC) and PubMed**, authors are allowed to post pre-prints in the PubMedCentral repository if the work was produced by NIH funded research and the paper has also been submitted to a peer-reviewed journal. No revisions are allowed to be made to the submission.

**FDA pilot website projects**

**2021. The COVID-19 EUA FAERS Public Dashboard** shows Adverse Events Related to COVID-19 Products. It is a public data dashboard showing human adverse event reports for drugs and therapeutic products used under emergency use authorization (EUA) during COVID-19. It provides weekly updates of adverse event reports submitted to FAERS.

## Supporting information

S1 Table 21^st^ Century datasets utilized in evaluating NM/R-associated COVID-19 rebound. (DOCX)
