# Supplementary material for: A SONAR report on Nirmatrelvir/ritonavir-associated rebound COVID-19: Using new databases for evaluating new diseases
Source: PLoS One. 2024 Sep 25;19(9):e0308205. doi: 10.1371/journal.pone.0308205 (PMC11423969; doi:10.1371/journal.pone.0308205)
Supplement: S1 Table — (DOCX) [file pone.0308205.s001.docx]

Supplemental Table 1. 21^st^ Century Datasets utilized in evaluating NM/R-associated COVID-19 rebound

| Database- start date of the database | # of users | # of cases reported on the specific web site | Completeness of individual case reports | Information on Omnicron versus Delta | Case information subsequently reported in the peer-reviewed literature | Descriptions of specific patients | Updated |
| --- | --- | --- | --- | --- | --- | --- | --- |
| Google -1998 | 246 million | 4 | Intermediate | None | NEJM-1 pt (the former director of NIAID) | Virologist, Executive in the Biden Administration, wife of the executive in the Biden Administration, CDC Executive, Host of a Television Talk Show | Every second |
| Facebook-2004 | 190 milion | 0 | Low | … | … | … | Every second |
| REDDIT-2005 | 52 million | 0 | Low | … | … | … | Every second |
| Twitter-2006 | 63 million | 3 | Moderately High | None | No | Wife of Chairman of Medicine at a medica school, Dean at a graduate school of tropic medicine, Oncology Associate Professor at a medical school | Every second |
| CDC-Twitter- 2008 | 5.3 million | 0 | …. | … | … | … | Daily |
| Instagram-2010 | 143 million | 0 | Low | … | … | … | Every second |
| FAERS Dashboard-2021 | Not known | 12,688 | Very low | Very little | Not known | … | Updated weekly; |
|  |  |  |  |  |  |  |  |
| Pre-print services | # of pre-prints |  | Quality of case information | Demographics | # of COVID-19 rebound patients | Information on individual patients w/ confirmed or suspected NM/r rebound | Where from |
| medRxiv- 2018 |  |  | High | No | 7 pts | 7 pts (6 confirmed and 1 presumed COVID-19 NM/r rebound | 7 pts from Mass General Hospital study |
| Research Square-2018 | 150,000 | 3 | High | Yes | 10 pts | 10 pts (3 confirmed and 7 presumed NM/r patients) | 2 from Columbia; 1 from New England VA; 7 from Colombia U study |
| NIH pre-print on PMC site – 2020 |  | 0 | NA | NA | NAs | NA | NA |
